# Sex‐related differential susceptibility to ponatinib cardiotoxicity and differential modulation of the Notch1 signalling pathway in a murine model

**DOI:** 10.1111/jcmm.17008

**Published:** 2022-02-05

**Authors:** Rosalinda Madonna, Damiana Pieragostino, Maria Concetta Cufaro, Piero Del Boccio, Angela Pucci, Letizia Mattii, Vanessa Doria, Christian Cadeddu Dessalvi, Riccardo Zucchi, Giuseppe Mercuro, Raffaele De Caterina

**Affiliations:** ^1^ Department of Pathology Institute of Cardiology University of Pisa Pisa Italy; ^2^ Department of Innovative Technologies in Medicine and Dentistry ‘‘G. d’Annunzio’’ University of Chieti‐Pescara Chieti Italy; ^3^ Department of Pharmacy ‘‘G. d’Annunzio’’ University of Chieti‐Pescara Chieti Italy; ^4^ Analytical Biochemistry and Proteomics Laboratory Center for Advanced Studies and Technology (CAST) "G. d'Annunzio" University of Chieti‐Pescara Chieti Italy; ^5^ Department of Histopathology Pisa University Hospital Pisa Italy; ^6^ Department of Clinical and Experimental Medicine University of Pisa Pisa Italy; ^7^ Institute of Cardiology, “G. D’Annunzio University of Chieti Pescara Italy; ^8^ Department of Medical Sciences and Public Health University of Cagliari Cagliari Italy; ^9^ Department of Pathology Laboratory of Biochemistry University of Pisa Pisa Italy; ^10^ Fondazione VillaSerena per la Ricerca Città Sant’Angelo Pescara Italy

**Keywords:** apoptosis, cardiotoxicity, DNA damage, mouse model, Notch1, ponatinib, sex‐based differences

## Abstract

Ponatinib (PON), a tyrosine kinase inhibitor approved in chronic myeloid leukaemia, has proven cardiovascular toxicity. We assessed mechanisms of sex‐related PON‐induced cardiotoxicity and identified rescue strategies in a murine model. PON+scrambled siRNA‐treated male mice had a higher number of TUNEL‐positive cells (%TdT+6.12 ± 0.17), higher percentage of SA‐β‐gal‐positive senescent cardiac area (%SA‐β‐gal 1.41 ± 0.59) and a lower reactivity degree (RD) for the survival marker Bmi1 [Abs (OD) 5000 ± 703] compared to female (%TdT+3.75 ± 0.35; %SA‐β‐gal 0.77 ± 0.02; Bmi1 [Abs (OD) 8567 ± 2173]. Proteomics analysis of cardiac tissue showed downstream activation of cell death in PON+siRNA scrambled compared to vehicle or PON+siRNA‐Notch1‐treated male mice. Upstream analysis showed beta‐oestradiol activation, and downstream analysis showed activation of cell survival and inhibition of cell death in PON+scrambled siRNA compared to vehicle or PON+siRNA‐Notch1‐treated female mice. PON+scrambled siRNA‐treated mice also had a downregulation of cardiac actin—more marked in males—and vessel density—more marked in females. Female hearts showed greater cardiac fibrosis than their male counterparts at baseline, with no significant change after PON treatment. PON+siRNA‐scrambled mice had less fibrosis than vehicle or PON+siRNA‐Notch1‐treated mice. The left ventricular systolic dysfunction showed by PON+scrambled siRNA‐treated mice (male %EF 28 ± 9; female %EF 36 ± 7) was reversed in both PON+siRNA‐Notch1‐treated male (%EF 53 ± 9) and female mice (%EF 52 ± 8). We report sex‐related differential susceptibility and Notch1 modulation in PON‐induced cardiotoxicity. This can help to identify biomarkers and potential mechanisms underlying sex‐related differences in PON‐induced cardiotoxicity.

## 1 INTRODUCTION

Tyrosine kinase inhibitors (TKIs) are a group of drugs capable of selectively inhibiting tyrosine kinases,^9^ the constitutive expression of which leads to de‐regulated cell proliferation and tumour formation.^3^ In chronic myelogenous leukaemia (CML), a reciprocal translocation (9; 22) (q34; q11), known as the ‘Philadelphia chromosome’ (Ph), results in the formation of a mutated tyrosine kinase protein, called Abelson‐Breakpoint Cluster Region (BCR/ABL). Ponatinib (PON, trade name: Iclusig, from Incyte Biosciences, Epalinges, Switzerland) is the only TKI capable of inhibiting BCR/ABL3, thus leading to growth arrest and apoptosis of the neoplastic clone.^17^ However, a significant number of patients treated with PON, as—in general—with all TKIs, develops vascular and cardiac toxicity, with evidence of congestive heart failure and electrocardiographic abnormalities.^39^ Although several mechanisms have been hypothesized for PON‐induced cardiotoxicity, the in vivo effects of PON on the heart have not been fully characterized.

Notch1 is a master regulator of B‐ and T‐cell development.^32^, ^34^ While in some types of leukaemia and T‐cell lymphoma the aberrant activation of the Notch1 pathway due to 5’ promoter deletions is associated with atypical myloproliferative disease,^36^ in human CML cells Notch1 signalling plays a tumour suppressive role, and its overexpression may inhibit tumour proliferation.^1^, ^43^ Therefore, targeting Notch1 may represent a therapeutic approach for CML. However, Notch1 plays also a central role in vascular development.^11^ The hyperactivation of Notch1 in vessels leads to abnormal vascular development and vascular dysfunction.^1^ We have previously shown that selective blockade of Notch1 can prevent PON‐induced vascular toxicity in human aortic endothelial cells.^24^ Therefore, activation of Notch1 in CML cells by PON can be considered as the ‘on‐target effect’ on the tumour.

The extent of PON‐induced cardiotoxicity is quite variable, and this is a confounding aspect for identifying its mechanisms.^6^, ^38^ In particular, little is known about the molecular mechanisms of PON‐induced cardiotoxicity and the possible differences between males and females. Such type of research can help to identify biomarkers and strategies to counteract PON‐induced cardiotoxicity. Here, we report a sex‐related differential susceptibility to PON‐induced cardiotoxicity and identify Notch1 as the differentially affected signalling pathway.

## 2 METHODS

### 2.1 Materials

Ponatinib (PON) was purchased from Incyte S.r.l (Wilmington, Delaware, US). SiRNA‐Notch1 and scrambled siRNA were purchased from Invitrogen Life Technologies (Carlsbad, California, US).

### 2.2 Animals and mice randomization

Male and female C57BL/6 mice (body weight: 30 ± 4 g, 24 months old) were purchased from Charles River Italia (Lecco, Italy). Mice were housed under a 12‐hour light/dark cycle (7 am‐7 pm) in temperature‐ and humidity‐controlled rooms and were provided with *ad libitum* rodent chow (Teklad 7001, 4.4%; Harlan Teklad Global Diets) and water. Animals were randomized into 3 groups (n=12, n=6 male and n=6 female for each treatment group): vehicle, PON +scrambled siRNA, PON +siRNA‐Notch1. PON was dissolved in DMSO and administered to mice in the experimental groups by oral gavage daily (30 mg/kg/d for 28 days, corresponding to the oral doses clinically used in humans; https://go.drugbank.com/drugs/DB08901), with siRNA‐Notch1 or scrambled siRNA administered via tail vein every 3 days. The vehicle group was given the same dosage of DMSO dissolved in the same volume of water for 28 days. For echocardiograms, mice were anaesthetized by intraperitoneal injection of ketamine (100 mg/kg, Chlorketam; Vétoquinol, Lure, France). The animals were then euthanized by deep anaesthesia under 2% isoflurane and cardiac puncture, and a terminal blood sample was immediately drawn from the left ventricle. The blood was centrifuged and serum stored at −80 °C until biochemical analyses. Hearts were excised, snap‐frozen in liquid nitrogen and stored at −80 °C for protein extraction or embedded in optical cutting temperature (OCT) medium and stored at −80 °C for histological analyses. All procedures were approved by the local Institutional Ethics Committee for Animal Research (Protocol number 176/2019‐R released on February 25 2019). All studies comply with the Guidelines from Directive 2010/63 EU of the European Parliament on the protection of animals used for scientific purpose.

### 2.3 Preparation of transit TKO‐siRNA complexes and siRNA delivery in vivo

Non‐viral siRNA delivery in vivo was done by tail vein injection. For monitoring transfection efficacy, we used scrambled siRNA conjugated with a Cy3 fluorochrome provided by Mirus (Mirus Bio, Madison, WI, USA). Accordingly, 1.33 μL (25 nM) of Cy3‐conjugated scrambled siRNA or Cy3‐conjugated Notch1‐siRNA (NM_008714.3) was combined with 0.5 μL of Transit‐TKO (Mirus) in a final volume 10 μL sterile H_2_O and incubated for 30 min at room temperature, according to a previously published protocol.^15^ According to mice randomization, tail vein injections of phosphate‐buffered saline (10 μL PBS, vehicle group, N = 6 C57BL/6 male mice and N = 6 C57BL/6 female mice), 10 μL scrambled siRNA (N = 6 C57BL/6 male mice and N = 6 C57BL/6 female mice) or 10 μL Notch1‐siRNA (N = 6 C57BL/6 male mice and N = 6 C57BL/6 female mice) were performed with a 32 gauge needle attached to a 1 mL syringe (Beckton Dickinson, Franklin Lake, New Jersey, US). Injections were repeated after 72 h and every three days from the first injection.

### 2.4 Tissue preparation and analysis of fibrosis

After induction of anaesthesia, the hearts were removed and embedded in OCT without fixation. The blocks from each heart were cut transversely to obtain 30 sections (each 5 μm thick) from the middle of the ventricles. All histological sections were analysed blindly. Total cardiac fibrosis (including interstitial, perivascular and coronary arterial fibrosis) was assessed by Masson's trichrome staining. Digital colour images were obtained, and the extent of left ventricular (LV) fibrosis was evaluated with the NIH Image J software (Media Cybernetics, Rockville, MD, USA, http://rsb.info.nih.gov/ij) in 5 cardiac areas randomly selected for each heart section and expressed as % of the total LV area.

### 2.5 Terminal Deoxyribonucleotidyl Transferase–mediated dUTP Nick End Labeling (TUNEL) Assay

Detection of nuclei with fragmented DNA by TUNEL was performed using the HRP‐DAB TUNEL assay kit (Abcam, Cambridge, UK) according to the manufacturer's instructions and a previously published protocol.^21^ Methyl green (DakoCytomation) was used as a counterstain. The myocardial apoptotic index was calculated as the number of positive cardiac cells for field/total cardiac cell number for field x 100.

### 2.6 Senescence‐Associated β‐galactosidase Assay

The effect of PON with/without siRNA‐Notch1 or scrambled siRNA on cardiac senescence was evaluated by SA‐b‐gal Activity Senescence‐Associated‐β‐Galactosidase Staining (Cell Biolabs, Inc, San Diego, CA, USA), as previously described.^24^ Cardiac frozen sections were post‐fixed in 4% paraformaldehyde at room temperature for 15 min, rinsed with sterile PBS and incubated overnight with fresh senescence‐associated β‐galactosidase staining solution (1 mg/mL X‐gal in 40 mM citric acid/sodium phosphate, pH 6.0, 5 mM potassium ferricyanide, 5 mM potassium ferrocyanide, 150 mM sodium chloride, 2 mM magnesium chloride) at 37 °C. Then, the staining solution was removed, sections were mounted in 70% glycerol, and the development of blue colour was assessed under standard light microscope. The extent of the blue‐stained area was evaluated with the Image J software in 5 randomly chosen cardiac areas per each heart section and expressed as % of total LV area.

### 2.7 Evaluation of Bmi1 expression

The 5 μm OCT sections were permeabilized, blocked for 60 min in PBS containing 0.25% BSA and 0.1% Tween and incubated overnight at 4 °C with a rabbit anti‐Bmi1 primary antibody (Thermo Fisher Scientific, IL, USA). A non‐immune IgG (Becton, Dickinson and Co., Franklin Lakes, New Jersey, USA) was used as the isotype control. After washing with PBS, cardiac sections were stained with an Alexa‐Fluor 568‐conjugated anti‐rabbit secondary antibody, washed, mounted, inspected under an immunofluorescence microscope and captured at 100x magnification with a digital camera (SC50, Olympus, Shibuya‐ku, Tokyo, Japan). The immunofluorescence of images was quantified with the CellSens Imaging Software (Olympus). Two microscopic fields, usually covering the whole section, were analysed for each sample. The mean colour threshold (the level above which images were considered to be reacting) was evaluated on negative controls. The image analysis was performed considering the percentage of the reacting area and the respective level of pixel colour intensity per field. Thus, the amounts of Bmi1 immunopositivity were expressed as a Bmi1 reactivity degree and calculated as the product between the average of positive area percentage and the mean value of pixel colour intensity per microscopic field.

### 2.8 Histological evaluation of sarcomeric organization and vessel density

For immunofluorescence staining of sarcomeres and arterioles, 5 μm OCT sections were permeabilized, blocked for 30 min in PBS containing 1% bovine serum albumin and incubated for 1 hour at 4 °C with a primary anti‐α‐smooth muscle actin (ASMA, Sigma Aldrich, St. Louis, MI, USA) or an anti‐sarcomeric α‐actinin (Sigma) antibody. A non‐immune IgG (Becton, Dickinson and Co., Franklin Lakes, New Jersey, USA) was used as isotype control. After washing with PBS, cardiac sections were stained with a fluorescein isothiocyanate (FITC)‐conjugated secondary antibody, washed, mounted and viewed under an immunofluorescence microscope. Arterioles were counted blindly in 5 randomly selected fields at 10× magnification. Vascular images were captured by using an inverted light microscope (Olympus IX71) and analysed by using Image J software. Vessel density was evaluated in 5 randomly chosen cardiac areas per each heart section and expressed as % of total LV area.

### 2.9 Proteomics and computational analyses

Cardiac tissue from each treatment group was digested following the Filter Aided Sample Preparation (FASP) method. To identify and simultaneously quantify proteins expressed in the hearts, label‐free shotgun proteomics experiments were carried out according to detailed protocols described in the Online Supplement. Mass spectrometry proteomics data are deposited to the ProteomeXchange Consortium via the PRIDE^31^ partner repository with PXD017413 data set identifier. A panel of differential proteins (considering only unique proteins) was subjected to an in‐silico analysis by the Ingenuity Pathway Analysis (IPA) (Ingenuity Systems, Mountain View, CA) and Gene Ontology.

### 2.10 Immunoblotting

To examine the effects of PON with/without siRNA‐Notch1 or scrambled siRNA on the levels of cardiovascular and fibrosis markers, total proteins from cardiac samples were isolated in an ice‐cold RadioImmuno Precipitation Assay (RIPA) lysis buffer containing 10 mmol/litre Tris, pH 7.4, 1% SDS and 1× protease inhibitor (1 mmol/litre sodium orthovanadate) (S8830, Sigma Aldrich, Merck KGaA, Darmstadt, Germany). After centrifugation at 15,000 rpm for 20 min at 4 °C, supernatants were collected and the protein concentration was evaluated by the bicinchoninic acid assay (BCA) (Pierce(BCThermo Fisher Scientific) microplate method. Proteins (15 μg/lane) were separated under reducing conditions (125 mm Tris, pH 6.8, 4% SDS, 10% glycerol, 0.006% bromphenol blue, 2% β‐mercaptoethanol) by electrophoresis onto 5 – 20% SDS‐polyacrylamide gel (Criterion gels from Biorad, Hercules, CA) and electroblotted to polyvinylidene fluoride membrane (Immobilon‐P, Millipore, Bedford, MA, USA). The membranes were reversibly stained with Ponceau red (Sigma) to verify equal protein loading and/or transfer. After blocking in Tris‐buffered saline (0.2 m Tris and 8% NaCl) containing 5% nonfat powdered milk and 0.1% Tween 20 for 1 h at room temperature, the membranes were incubated overnight at 4 °C with the following primary antibodies to (1) anti‐cardiac actin (Sigma), (2) anti‐metalloproteinase type 8 (Santa Cruz Biotechnology, Dallas TX), (3) anti‐aquaporin isoform 1 (Santa Cruz Biotechnology), (4) anti‐FLT‐1 (Santa Cruz Biotechnology) and (5) anti‐collagen type‐1 (Sigma). The blots were incubated with horseradish peroxidase‐coupled secondary antibodies diluted in 5% nonfat powdered milk, washed and developed using a SuperSignal West Pico Chemiluminescent Substrate Kit (Pierce). The intensity of each immunoreactive protein band was quantified by densitometric analysis using the Image Lab Software (BioRad). Equal loading/equal protein transfer was verified by stripping and reprobing the blots with anti‐GAPDH (Abcam).

### 2.11 Echocardiography

We performed transthoracic echocardiography 1 month after treatments to assess the functional effects of each treatment using a portable ultrasound apparatus (Esaote; Genoa, Italy) equipped with a 21‐MHz linear probe according to detailed protocols described in the Online Supplement.

### 2.12 Statistical Analysis

Data are expressed as mean ±standard deviation (SD). Two‐group comparisons were performed by using the Student's *t* test for unpaired values. Multiple‐group comparisons were performed using analysis of variance and the Gabriel or Tukey Honestly Significant Difference (HSD) post‐hoc test to determine statistical significance within and between groups. *P*‐values <0.05 were considered statistically significant.

## 3 RESULTS

### 3.1 Ponatinib induces differential apoptosis, senescence and expression of the survival marker Bmi1 in male and female hearts

**FIGURE 1:**
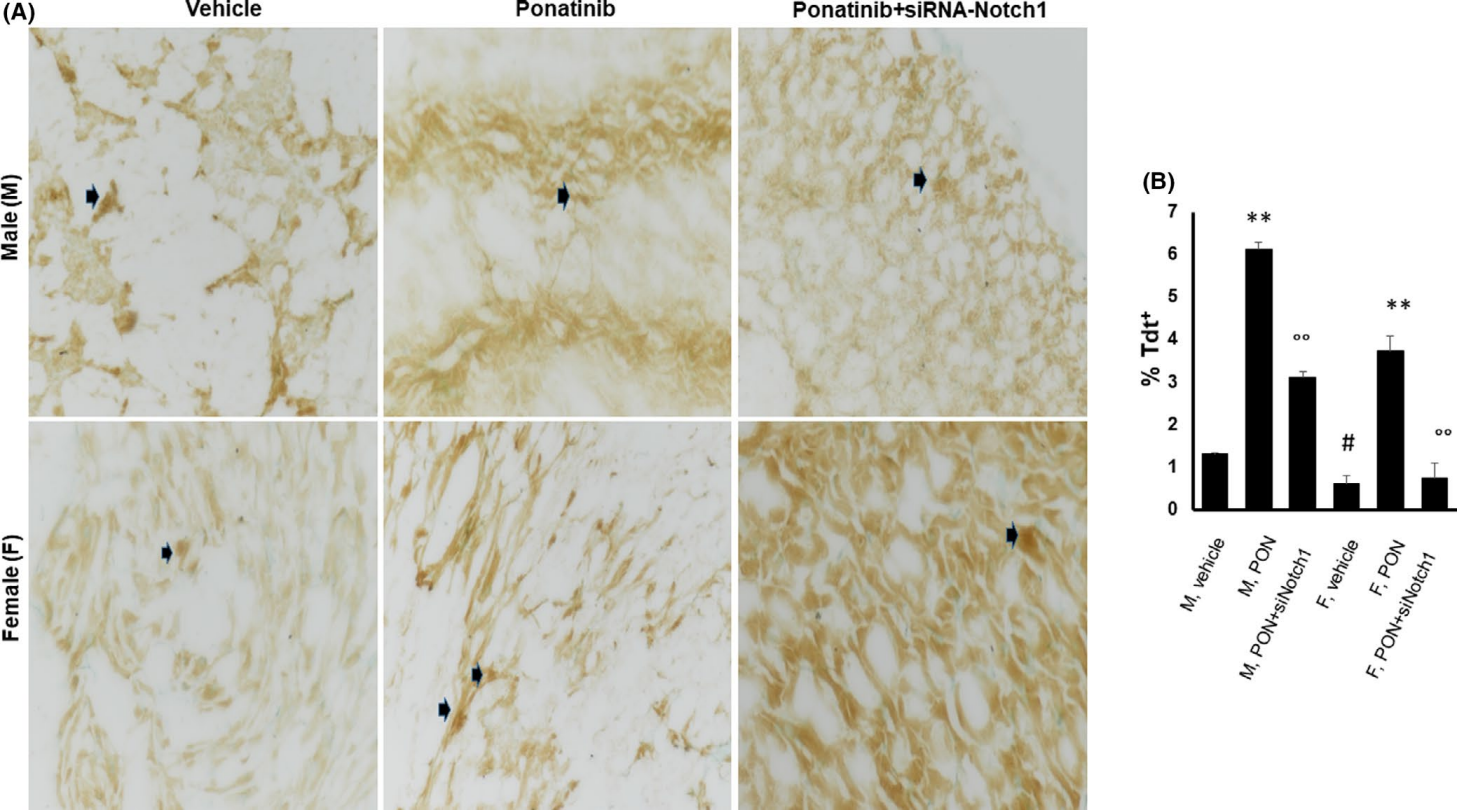
Effects of ponatinib and Notch‐1 signalling inhibition on cardiac apoptosis in male and female mice. Detection of total apoptotic cells in male and female hearts of mice treated with vehicle (n = 3) or PON+scrambled siRNA (n=3) or PON+siRNA‐Notch1 (n=3) by TUNEL assay (A) and apoptotic index (B). Values are expressed as means ±standard deviations. ** vs vehicle, *p* <0.01; * vs vehicle, *p* <0.05; °° vs PON, *p* <0.01; # vs M vehicle, *p* <0.05. Arrows indicate apoptotic cells. Magnification 40x. Abbreviations: PON, ponatinib; M, male; F, female

We examined whether there is evidence of apoptosis and senescence of cardiac cells in our ponatinib‐induced cardiotoxicity model and whether there is a differential effect according to sex. In the ventricles of PON+scrambled siRNA mice, the total number of TUNEL‐positive cells was fivefold and threefold higher in males and females, respectively, than in vehicle‐treated controls, with a statistically significant sex‐related difference. Indeed, male mice featured twofold greater numbers of TUNEL‐positive cells than females (Figure 1A and B). These effects were reversed by co‐treatment with siRNA‐Notch1, suggesting that PON acts on cardiac apoptosis differently in both sexes via the Notch‐1 signalling pathway (Figure 1A and B).

**FIGURE 2:**
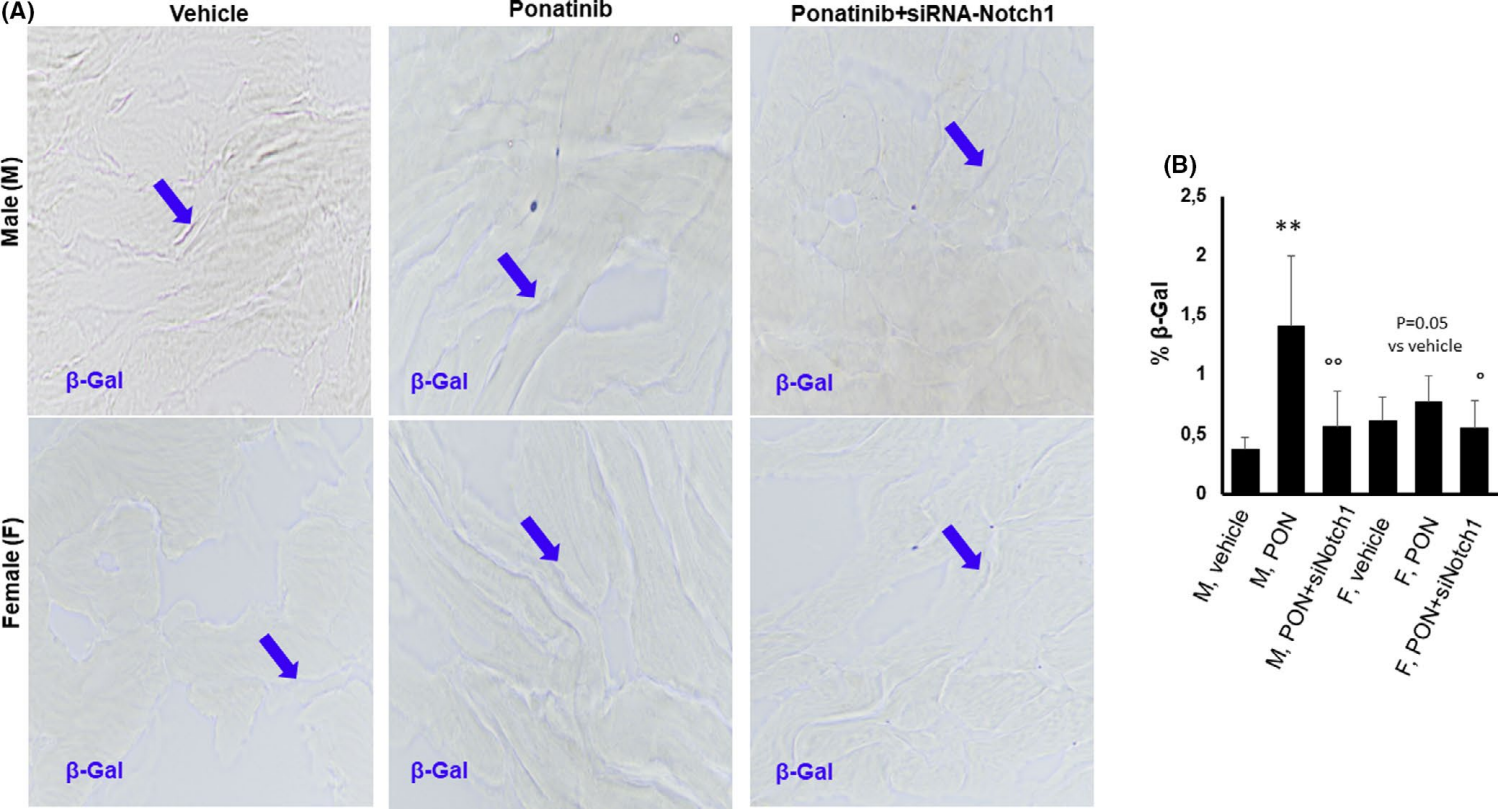
Effects of ponatinib and Notch‐1 signalling inhibition on cardiac senescence‐associated β‐galactosidase activity in male and female mice. Detection of senescence in male and female hearts of mice treated with vehicle (n = 5) or PON+scrambled siRNA (n=5) or PON+siRNA‐Notch1 (n=5) by senescence‐associated β‐galactosidase (SA β‐Gal) staining (A) and quantitative evaluations (B) in cardiac tissue. Data are expressed as means ±standard deviations. ** vs vehicle, *p* <0.01; °° vs PON, *p* <0.01; ° vs PON, *p* <0.05. Arrows indicate senescent areas. Magnification 40x. Abbreviations: PON, ponatinib; M, male; F, female

Senescence was identified by the expression of senescence‐associated β‐galactosidase (SA β‐gal). In the ventricular myocardium of PON+scrambled siRNA mice, the percentage of SA β‐gal‐ positive senescent cardiac area was onefold higher in male mice than in vehicle‐treated controls (Figure 2A, B). Female mice, however, exhibited a constant SA β‐gal‐positive senescent cardiac area with no statistically significant PON effect compared to vehicle‐treated controls (Figure 2A, B). The effect of PON on cardiac senescence was reversed by co‐treatment with siRNA‐Notch1, which is in line with the results obtained with TANEL staining (Figure 2A, B).

**FIGURE 3:**
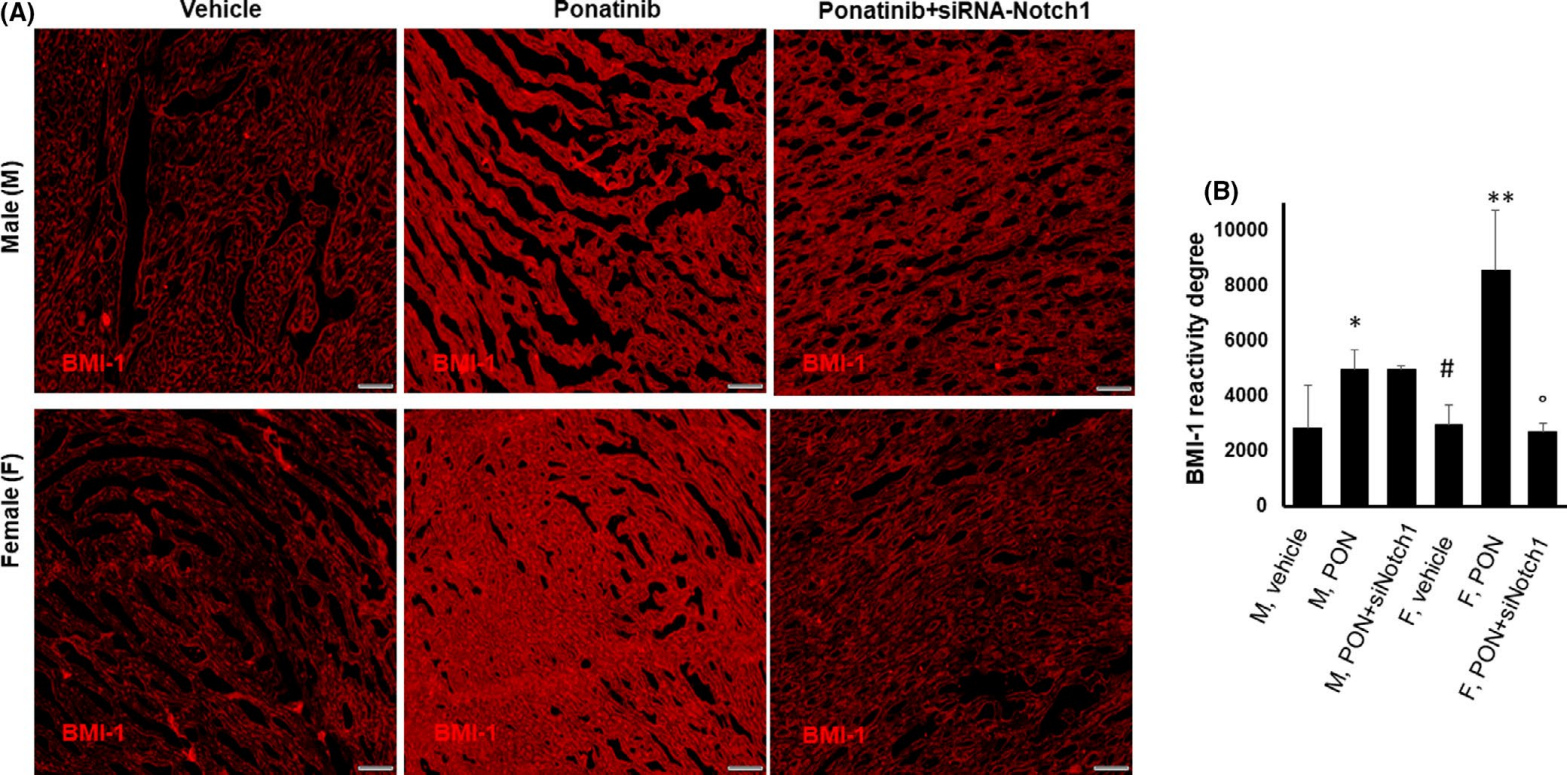
Effects of ponatinib and Notch‐1 signalling inhibition on endogenous cardiac Bmi1 expression in male and female mice. Detection of Bmi1 in male and female hearts of mice treated with vehicle (n = 5) or PON+scrambled siRNA (n=5) or PON+siRNA‐Notch1 (n=5) by immunofluorescence staining (A) and quantitative evaluations (B) in cardiac tissue. Data are expressed as means ±standard deviations. ** vs vehicle, *p* <0.01; * vs vehicle, *p* <0.05; ° vs PON, *p*<0.05; # vs M vehicle, *p*<0.05. Magnification 40x. Abbreviations: PON, ponatinib; M, male; F, female

Immunohistochemical staining showed a prominent effect of PON in female mice with a fourfold increase in expression of the Bmi1 prosurvival marker compared to vehicle, while the increase was mild in the male counterpart (Figure 3A, B). In female mice, these effects were almost totally reversed by co‐treatment with siRNA‐Notch1, suggesting that PON acts on endogenous cardiac expression of Bmi1 differently in both sexes via the Notch‐1 signalling pathway.

### 3.2 Ponatinib induces differential myofibre structure disruption and cardiac actin reduction in male and female hearts

**FIGURE 4:**
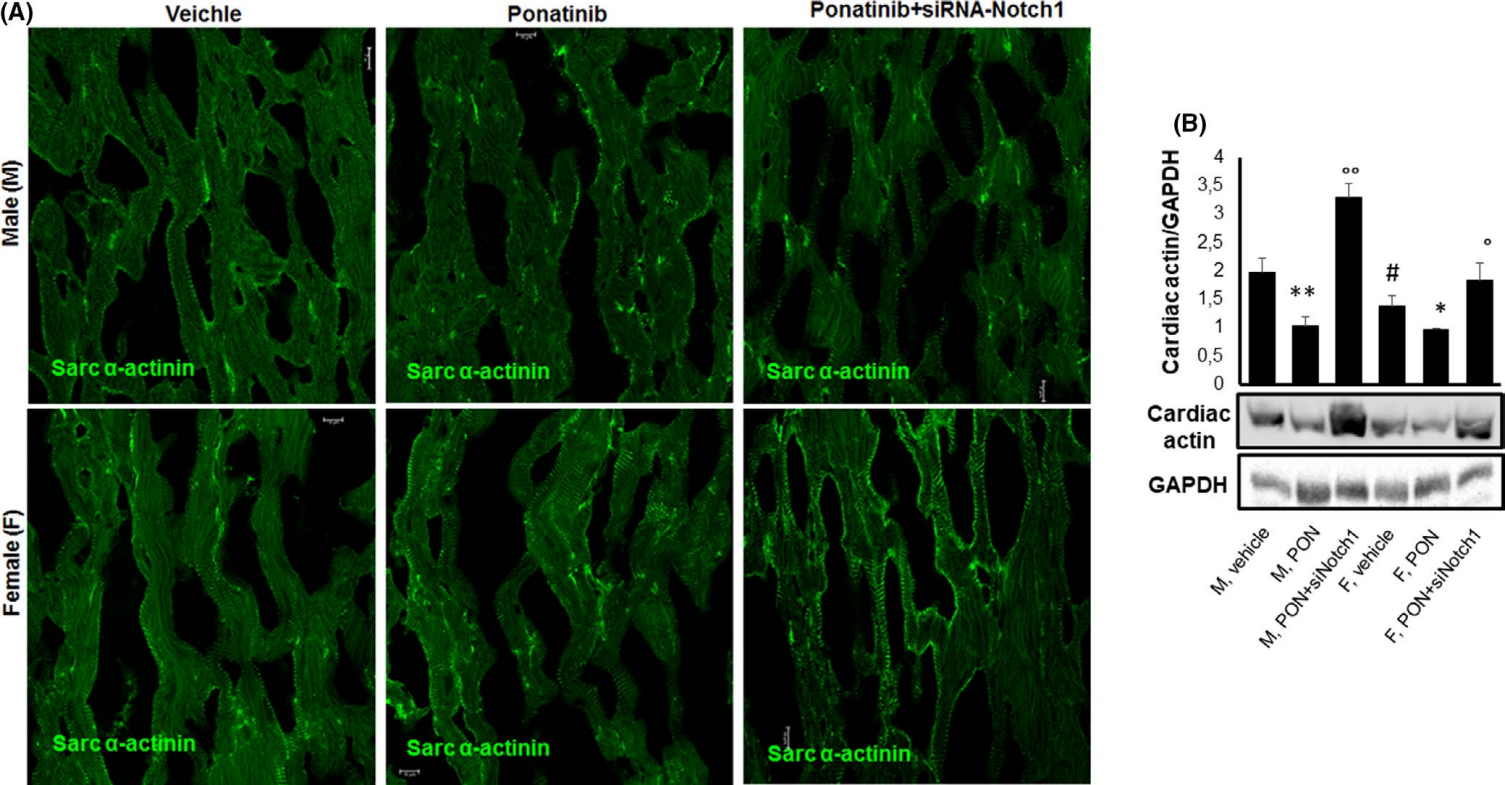
Effects of ponatinib and Notch‐1 signalling inhibition on myofibre organization and cardiac actin expression in male and female mice. Detection of (A) sarcomeric α‐actinin by immunofluorescence staining (n=5) and (B) cardiac actin by immunoblotting (n=4) in male and female hearts of mice treated with vehicle or PON+scrambled siRNA or PON+siRNA‐Notch1. Quantitative evaluations of cardiac actin are expressed as means ±standard deviations. ** vs vehicle, *p* <0.01; * vs vehicle, *p* <0.05; ° vs PON, *p* <0.05; °° vs PON, *p* <0.01; # vs M vehicle, *p* <0.05. Magnification 40x. Abbreviations: PON, ponatinib; M, male; F, female. The GAPDH blot shown in the figure is representative of the control carried out also for the blots shown in Figures 6 and 7

We monitored the anti‐cardiac alpha‐actinin immune reactivity to ascertain the impact of PON on myofibre structure (Figure 4A). A well‐organized sarcomeric structure was evident in the hearts of vehicle‐treated male and female mice. In contrast, myofibre structure was disrupted in the hearts of male mice treated with from PON+scrambled siRNA, as evidenced by the widespread reduction of sarcomeric staining. This effect was accompanied by the expression of onefold lower cardiac actin levels in PON+scrambled siRNA‐treated compared to vehicle‐treated male mice (Figure 4B). Disruption of myofibre structure was less pronounced in the hearts of PON+scrambled siRNA‐treated female mice (Figure 4A), which also showed less marked reduction of cardiac actin expression than in vehicle‐treated female mice (Figure 4B). The sarcomeric structure was reorganized, as well as the downregulation of cardiac actin was reversed in the hearts of male and female mice treated with PON+siRNA‐Notch1 (Figure 4A, B).

### 3.3 Proteomics analysis reveals the differential up‐ and downregulation of cell death and survival in male and female hearts exposed to ponatinib

**FIGURE 5:**
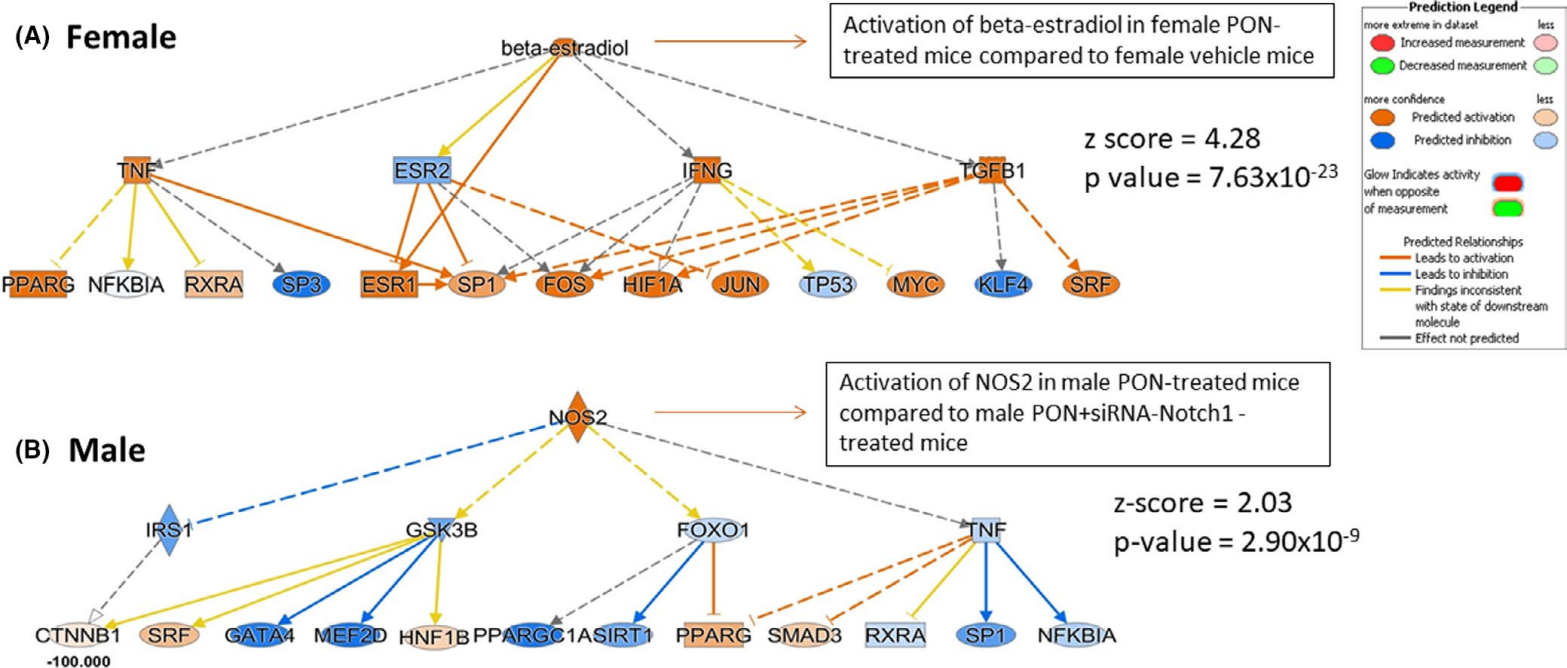
Upstream network analysis of male and female mice exposed to ponatinib and Notch‐1 signalling inhibition. Panel A‐B: The image shows upstream activation of the β‐oestradiol gene (A) in female PON‐treated compared to female vehicle‐treated mice, resulting in a z‐score of 4.28, and the upstream activation of inducible nitric oxide synthase (NOS2) gene (B) in male PON‐treated compared to male PON+siRNA‐Notch1‐treated mice, resulting in a z‐score of 2.03 respectively. Blue colour keys indicate inhibited regulators, while orange ones indicate the activated regulators. The intensity of colours is proportional to the score prediction value. z scores >2.0 indicate that a molecule and/or function is activated

We carried out label‐free proteomics analysis in the hearts of male and female mice to identify the expressional signatures of hearts exposed to PON+scrambled siRNA compared to those treated with vehicle or PON+siRNA‐Notch1. In particular, we obtained quantitative proteomics data through the MaxQuant software using label‐free quantitation (LFQ) intensity as a quantification parameter for individual protein expression. As reported in Methods, we quantified 476 proteins in vehicle‐treated female hearts, 847 proteins in PON+siRNA‐scrambled female hearts, 856 proteins in PON+siRNA‐Notch1 female hearts (Figure 1A), 657 proteins in vehicle vehicle‐treated male hearts, 803 proteins in PON+siRNA‐scrambled‐treated male hearts and 800 proteins in PON+siRNA‐Notch1‐treated male hearts (Figure 1B). The list of quantified ad differential expressed proteins in each group of mice is reported in the ProteomeXchange Consortium via the PRIDE^31^ partner repository. As reported in the volcano plot, 109 and 88 of these proteins were significantly downregulated and upregulated, respectively, in PON+scrambled siRNA‐treated female hearts compared to vehicle (Figure 2A), while 123 and 135 proteins were significantly downregulated and upregulated, respectively, in female hearts treated with PON+scrambled siRNA compared with PON+siRNA‐Notch1 (Figure 2B) (p‐value ≤0.05). In contrast, 34 and 38 proteins were significantly downregulated and upregulated, respectively, in PON+scrambled siRNA‐treated male hearts compared with vehicle (Figure 3A), while 39 and 38 proteins were significantly downregulated and upregulated in male hearts treated with PON+scrambled siRNA compared with PON+siRNA‐Notch1 (Figure 3B) (p‐value ≤0.05). We uploaded quantitative raw data for each different comparison in the Ingenuity Pathway Analysis (IPA) software in order to perform a Core Analysis. The main differentially activated and inhibited diseases and biofunctions in female and male hearts treated with vehicle or PON+scrambled siRNA compared to those co‐treated with PON+siRNA‐Notch1 are listed in Table 1 as a heatmap visualization of predictive z‐score generated by IPA. In detail, the orange colour indicates the predicted activation (z‐score ≥2.00), while the blue one highlights the predicted inhibition (z‐score ≤‐2.00). The colour intensity is directly proportional to the significance of the predicted activation or inhibition. As reported in Table 1, the protein cargo of female hearts treated with PON+scrambled siRNA was able to trigger more specific cellular processes linked to the activation of ‘cell survival’ (p‐value =1.38x10^−12^, z‐score =2.29) and ‘cell viability’ (p‐value =7.28x10^−12^, z‐score =2.32), whereas cellular functions related to ‘apoptosis’ (p‐value =7.85x10^−20^, z‐score = −3.11) and ‘apoptosis of muscle cells’ (p‐value =3.52x10^−10^, z‐score = −2.41) were significantly inhibited. On the other hand, the protein cargo of male hearts treated with PON+scrambled siRNA was able to trigger more cellular functions related to activation of ‘necrosis of muscle’ (p‐value =1.81x10^−17^, z‐score =2.23) and ‘cell death muscle cells’ (p‐value =2.16x10^−4^, z‐score =2.28). Moreover, as shown in Figure 4, the protein cargo of male hearts treated with PON+scrambled siRNA was able to significantly activate the ‘production of reactive oxygen species’ (p‐value =1.15x10^−32^, z‐score =2.14) together with their ‘synthesis’ (p‐value =2.88x10^−^25, z‐score =3.12) and ‘metabolism’ (p‐value =2.42x10^−^29, z‐score =2.75) compared to male mice treated with PON+siRNA‐Notch1. These data show the sex‐related differential susceptibility to PON‐induced cardiotoxicity, which could be reversed by the Notch1 knockdown. We used differential proteins obtained from male and female hearts treated with PON+scrambled siRNA or vehicle or PON+siRNA‐Notch1 for the Upstream Regulator Analysis by IPA. Most significant were the β‐oestradiol gene in female hearts treated with PON+scrambled siRNA compared to vehicle (p‐value =1.16x10^−23^, z‐score =3.4) (Figure 5A) and the inducible nitric oxide synthase (NOS2) gene in male hearts treated with PON+scrambled siRNA compared to PON+siRNA‐Notch1 (p‐value =2.93x10^−9^, z‐score =2.03) (Figure 5B). These data could further confirm that PON acts on the heart via the Notch‐1 signalling pathway and that both NOS2 and β‐oestradiol are differentially involved in the negative regulation of this pathway.

**TABLE 1:** Differentially activated and inhibited diseases and biofunctions

**Diseases and Biofunctions**	**Female**	**Male**
Cell survival	2.281	0.5
Cell viability	2.324	0.296
Necrosis of muscle	−1.289	2.232
Apoptosis of muscle cells	−2.409	1.378
Apoptosis	−3.112	−0.291
Necrosis of muscle	−1.289	2.232
Cell death of muscle cells	−1.31	2.28
Production/Synthesis of reactive oxygen species	−0.388	2.137
Concentrations reached of reactive oxygen species	0.106	3.12
Metabolism of reactive oxygen species	0.31	2.754

Note

Differentially activated and inhibited diseases and biofunctions in female and male hearts treated with PON+siRNA‐scrambled compared to those co‐treated with PON+siRNA‐Notch1. Table reports the predictive z‐score (orange activation z‐score ≥2.00; blue inhibition, z‐score ≤ −2.00). Concentrations reached of reactive oxygen species (ROS) refers to the final output of reactive oxygen species (ROS) that is the result of synthesis/production and degradation.

### 3.4 Ponatinib exerts anti‐angiogenic effect in male and female hearts via blocking Notch1 signalling pathway

**FIGURE 6:**
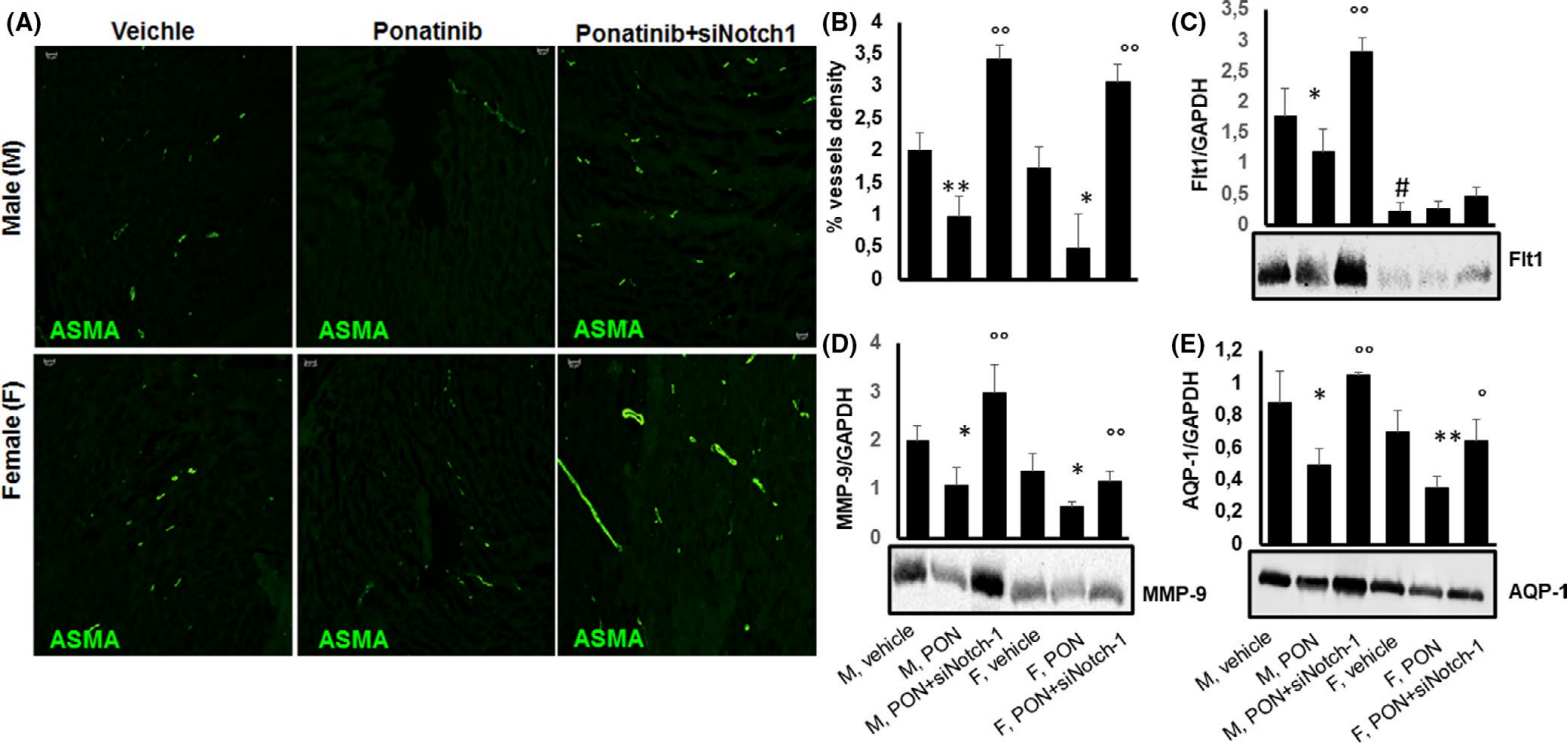
Effects of ponatinib and Notch‐1 signalling inhibition on vessel density and expression of angiogenic markers in male and female mice. Detection of (A, B) α‐smooth muscle actin (ASMA) by immunofluorescence staining (n=5), (C) Flt1 (n=4), (D) metalloproteinase (MMP)‐9 (n=5) and (E) aquaporin (AQP)‐1 (n=5) by immunoblotting in male and female hearts of mice treated with vehicle or PON+scrambled siRNA or PON+siRNA‐Notch1. Blot images are representative of a single experiment in which all the different antibodies have been probed against the tested markers on a single membrane. The quantitative data of immunoblotting are from the average of n=5 (MMP‐9 and AQP‐1) or n=4 (Flt1) independent experiments carried out on different membranes. Quantitative evaluations of vessel density and expression of Flt1, MMP‐9 and AQP1 are expressed as means ±standard deviations. ** vs vehicle, *p* <0.01; * vs vehicle, *p* <0.05; ° vs PON, *p* <0.05; °° vs PON, *p* <0.01; # vs M vehicle, *p* <0.05. Abbreviations: PON, ponatinib; M, male; F, female. Magnification 20x

Angiogenesis and vasculogenesis involve various angiogenic growth factor receptors with tyrosine kinase activity, such as vascular endothelial growth factor receptor‐1 (VEGFR‐1 or Flt1), fibroblast growth factor receptor (FGFR), platelet‐derived growth factor receptor (PFGR)^40^ and matrix metalloproteinases,^19^ all of which are targets of PON inhibitory tyrosine kinase activity. In addition, among angiogenic markers, the water channel aquaporin (AQP)‐1 expressed in peripheral vascular endothelial cells is involved in physiological and pathological angiogenesis,^25^, ^26^ as well as tumour metastasis and progression.^37^ Therefore, we evaluated the impact of PON on angiogenesis and the expression of angiogenic markers in male and female mice. Compared to vehicle‐treated mice, PON+scrambled siRNA showed a significant reduction in vessel density and expression of MMP‐9 and AQP‐1 (**Figure** **6** **A, B**) with no statistically significant differences between female and male mice. Female mice, however, showed constantly lower expression of Flt1 then male counterparts, with no statistically significant PON effect compared to vehicle‐treated controls (Figure 6A, B). The effects of PON were reversed by PON‐siRNA‐Notch1 co‐treatment, suggesting that PON acts on angiogenesis via the Notch1 signalling pathway (Figure 6A, B).

### 3.5 Ponatinib exerts anti‐fibrotic effect in male and female hearts independently from Notch1 signalling pathway

**FIGURE 7:**
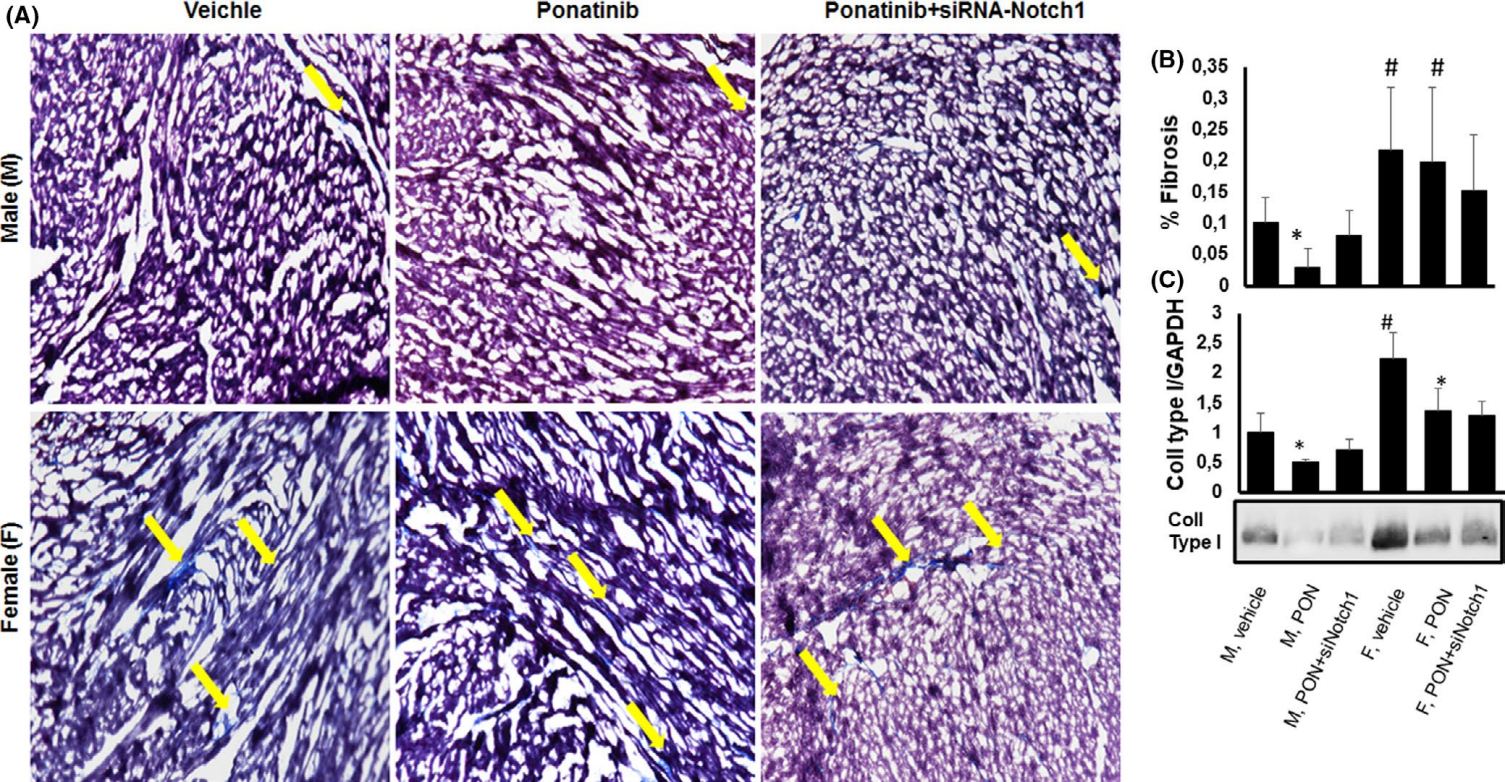
Effects of ponatinib and Notch‐1 signalling inhibition on cardiac fibrosis and collagen type 1 expression in male and female mice. Detection of (A, B) collagen deposition by Masson's Trichrome staining (n=5) and (C) collagen type 1 by immunoblotting (n=4) in male and female hearts of mice treated with vehicle or PON+scrambled siRNA or PON+siRNA‐Notch1. Quantitative evaluations of fibrosis and collagen type 1 expression are expressed as means ±standard deviations. * vs vehicle, *p* <0.05; # vs M vehicle, *p* <0.05. Magnification 40x. Abbreviations: PON, ponatinib; M, male; F, female

Cardiac remodelling in aged hearts is a multifaceted process that includes activation of fibroblasts and a complex immune response.^27^ In our experiments, total fibrosis was lower in vehicle‐treated male mice than in female counterparts, which confirms previous observations,^12^ and PON further attenuated collagen deposition (Figure 7A, B). This decrease in fibrosis was paralleled by a decrease in type I collagen expression (Figure 7C). There were no significant changes in collagen deposition and type I collagen expression after co‐treatment with PON+siRNA‐Notch1 (Figure 7A‐C), suggesting that Notch1 is not a direct mediator of the inhibitory effect of PON on fibrosis.

### 3.6 Ponatinib induces systolic but not diastolic dysfunction in female and male mice

To study the impact of PON on left ventricular (LV) systolic and diastolic function, we assessed LV ejection fraction (LVEF) and the E/A ratio by transthoracic echocardiography. Compared to vehicle, PON+scrambled siRNA induced 2.4‐fold and 1.4‐fold reduction in systolic function in male and female mice, respectively, as represented by a reduction in LVEF, with statistically significant sex‐related difference (Figure 5A). Co‐treatment with PON+siRNA‐Notch significantly attenuated these changes (Figure 5A). Diastolic dysfunction was more accentuated in vehicle‐treated female mice then in male counterparts, as represented by a lower E/A ratio, and PON did not affect diastolic dysfuntion, which parallels previous observations on cardiac fibrosis (Figure 5B).

## 4 DISCUSSION

In the present study, we show a sex‐related different susceptibility to PON‐induced cardiotoxicity, higher in male than female mice, as revealed by (1) higher numbers of TUNEL‐positive cells, (2) higher percentage of SA β‐gal‐positive senescent cardiac areas, (3) lower expression of the survival marker Bmi1, (4) more pronounced disruption of myofibre structure, (5) predicted downstream activation of cell death and inhibition of cell survival and (6) more pronounced LV systolic dysfunction in male mice than in female counterparts. Both males and females showed decreased vessel density and downregulation of angiogenesis markers in response to PON treatment in the absence of statistically significant inter‐sex differences. Furthermore, at baseline and regardless of PON treatment, female mice exhibited greater cardiac fibrosis, associated with greater diastolic dysfunction.

The TUNEL assay is commonly used to detect DNA fragmentation, resulting from apoptotic signalling cascades.^23^ We here demonstrate sex‐based differences in the total number of TUNEL‐positive cells and in the percentage of senescent areas in the myocardium of PON‐treated mice, with consistently higher levels in male than in female mice. A number of in vitro studies indicate that PON mediated cardiomyocyte apoptosis, as evaluated by TUNEL assay, and suggested that cardiomyocyte loss plays a critical role in the development of cardiotoxicity.^30^, ^41^, ^42^ So far, no studies have examined the effect of PON on cardiac cell senescence. The present study is therefore the first to demonstrate sex‐based differential upregulation of the senescent and apoptotic pathways in response to PON treatment.

Bmi1 is a prosurvival marker with an important role in the response to DNA damage,^13^, ^16^ as well as in mitochondrial function and ROS homeostasis.^8^ Upregulation of endogenous cardiac Bmi1 expression is considered a biomarker of cardiac repair in response to injury. Its expression levels correlate inversely with cellular senescence,^28^ and its overexpression in H9c2 cells significantly reduced doxorubicin‐induced apoptosis.^10^ Here, immunohistochemical staining showed a prominent effect of PON in inducing expression of endogenous Bmi1 in female mice, while this increase was mild in male counterparts. Collectively, these findings suggest a greater susceptibility of male compared to female mice to PON‐induced cardiotoxic damage through a lesser activation of tissue repair pathways. This, along with the demonstration of PON‐induced upregulation of the senescent and apoptotic pathways, may further advance understanding of the mechanisms of cardiotoxicity and may help explain a higher degree of cardiac dysfunction and a lower %EF in PON‐treated male compared to female mice.

Heart tissue expresses both functional androgen and oestrogen receptors, suggesting a likely role for sex hormones in regulating cardiac function.^2^ Oestrogens exert cardioprotective action through the prevention of cardiomyocyte apoptosis and oxidative stress in response to ischaemia/reperfusion injury and anthracycline damage.^5^, ^29^ The cardioprotective effect of oestrogens is also achieved in the presence of comorbidities including arterial hypertension, through the activation of the PI3K‐Akt pathway and Notch signalling.^44^ The ability of oestrogens to modulate the Notch signalling pathway has also been extensively demonstrated in endothelial cells. In an in vitro model of human umblical endothelial cells (HUVEC), oestrogens exert a vasculoprotective and proangiogenic effect by activating Notch‐1 and Notch‐4 and deactivating Notch‐2.^45^ On the other hand, sex hormones influence the effect of antineoplastic drugs on the heart and peripheral tissues in general, acting on their pharmacokinetics. Specifically, PON is mainly metabolized by the hepatic cytochrome P450 CYP3A4, which is influenced by oestrogens.^18^ Oestrogens also reduce the extrahepatic expression of cytochrome CYP1A1, which is known to regulate TKI efflux from cardiomyocytes.^20^ Clinical studies and guidelines have identified sex as a risk factor for PON‐induced cardiovascular toxicity in humans.^7^, ^38^ Contrary to these findings, less severe myocardial apoptosis and cardiac dysfunction were reported for adult male mice (9 to 12 months old) treated with sunitinib compared to female counterparts.^15^ Although we did not measure female sex hormones in the present study, discrepancies with our study can be attributed to the different type of TKIs and animal models, since we tested PON in 24 months old female mice, which may have affected oestrogen levels.

In this study, we also demonstrated that PON exerts its cardiotoxic effects through the Notch1 pathway. Notch‐1 silencing prevented PON‐induced cardiac apoptosis and senescence, as well as vascular toxicity. Notch signalling is a conserved evolutionary pathway with a key role in cell proliferation, survival and differentiation, including development of the cardiovascular system and differentiation of the hematopoietic system. Notch1 involves a bi‐molecular interaction between receptor and ligand: upon activation of the Notch1 receptor by proteolytic cleavage; the Notch1 intracellular domain then translocates into the nucleus where it binds to CSL (CBF1, Suppressor of Hairless, Lag‐1),^33^ converting it from a repressor to a transcription activator.^4^ This can result in either tumour suppressive or tumorigenic function, depending on biological contexts^35^ and levels of Notch1 expression.^14^ Overexpression of the active form of Notch1 or Notch2 in the human KCL‐22 leukaemia cell line has been reported to inhibit proliferation, accompanied by an increase in Hes1 mRNA levels.^22^ In vessels, PON induces vasculotoxicity by activating Notch1, which can be considered as the ‘on‐target off tumour effect’ of this drug.^24^ Our data here demonstrate that silencing of the PON ‘on‐target off tumour’ effect through Notch receptor blockade leads to reversal of PON‐induced cardiotoxicity. Thus, PON induces cardiotoxicity via the Notch1 signalling pathway. Strategies for cardiac‐specific inhibition of Notch‐1 are warranted to protect the heart from PON‐induced cardiotoxicity, without interfering with the antitumour effect of the drug.

We acknowledge some limitations of our study such as the low number of mice per group, due to the higher mortality in the PON‐treated groups of mice, and the short follow‐up limited to 1 month.

In conclusion, we identified a sex‐related different susceptibility to PON‐induced cardiotoxicity in old C57BL/6 mice and delineated Notch1 as the signalling pathway for PON‐induced cardiotoxicity in male and female mice. This may help to identify biomarkers and potential mechanisms underlying sex‐related differences in PON‐induced cardiotoxicity, which might enable significant clinical improvements in the effectiveness of PON therapies.

## CONFLICTS OF INTEREST

The authors declare no conflict of interest.

## AUTHOR CONTRIBUTION

**Rosalinda Madonna:** Conceptualization (equal); Data curation (equal); Formal analysis (equal); Funding acquisition (equal); Methodology (equal); Validation (equal); Writing‐original draft (equal); Writing‐review & editing (equal). **Damiana Pieragostino:** Data curation (equal); Formal analysis (equal); Methodology (equal); Writing‐review & editing (equal). **Maria Concetta Cufaro:** Data curation (equal); Formal analysis (equal); Methodology (equal). **Piero Del Boccio:** Formal analysis (equal); Supervision (equal); Validation (equal); Writing‐review & editing (equal). **Angela Pucci:** Data curation (equal); Formal analysis (equal); Methodology (equal). **Letizia Mattii:** Conceptualization (equal); Formal analysis (equal); Methodology (equal); Supervision (equal); Writing‐review & editing (equal). **Vanessa Doria:** Data curation (equal); Methodology (equal). **Christian Cadeddu Dessalvi:** Data curation (supporting); Writing‐review & editing (equal). **Riccardo Zucchi:** Funding acquisition (equal); Project administration (equal); Supervision (equal); Writing‐review & editing (equal). **Giuseppe Mercuro:** Conceptualization (equal); Funding acquisition (equal); Supervision (equal); Writing‐review & editing (equal). **Raffaele De Caterina:** Funding acquisition (equal); Supervision (equal); Writing‐original draft (equal); Writing‐review & editing (equal).

## Supporting information

Fig S1

Fig S2

Fig S3

Fig S4

Fig S5

Supplementary Material

## Data Availability

Data available on request from the authors.

## References

[1] Balistreri CR , Madonna R , Melino G , Caruso C . The emerging role of Notch pathway in ageing: Focus on the related mechanisms in age‐related diseases. Ageing Res Rev. 2016;29:50‐65. 10.1016/j.arr.2016.06.004 27328278

[2] Bell JR , Bernasochi GB , Varma U , Raaijmakers AJ , Delbridge LM . Sex and sex hormones in cardiac stress–mechanistic insights. J Steroid Biochem Mol Biol. 2013;137:124‐135. 10.1016/j.jsbmb.2013.05.015 23770428

[3] Blume‐Jensen P , Hunter T . Oncogenic kinase signalling. Nature. 2001;411:355‐365. 10.1038/35077225 11357143

[4] Bray SJ . Notch signalling: a simple pathway becomes complex. Nat Rev Mol Cell Biol. 2006;7:678‐689. 10.1038/nrm2009 16921404

[5] Cadeddu Dessalvi C , Pepe A , Penna C . Sex differences in anthracycline‐induced cardiotoxicity: the benefits of estrogens. Heart Fail Rev. 2019;24:915‐925. 10.1007/s10741-019-09820-2 31256318

[6] Caocci G , Mulas O , Annunziata M . Long‐term mortality rate for cardiovascular disease in 656 chronic myeloid leukaemia patients treated with second‐ and third‐generation tyrosine kinase inhibitors. Int J Cardiol. 2020;301:163‐166. 10.1016/j.ijcard.2019.10.036 31711851

[7] Caocci G , Mulas O , Abruzzese E . Arterial occlusive events in chronic myeloid leukemia patients treated with ponatinib in the real‐life practice are predicted by the Systematic Coronary Risk Evaluation (SCORE) chart. Hematol Oncol. 2019;37:296‐302. 10.1002/hon.2606 30892724 PMC6766852

[8] Chatoo W , Abdouh M , David J . The polycomb group gene Bmi1 regulates antioxidant defenses in neurons by repressing p53 pro‐oxidant activity. J Neurosci. 2009;29:529‐542. 10.1523/JNEUROSCI.5303-08.2009 19144853 PMC2744209

[9] Di Lisi D , Madonna R , Zito C . Anticancer therapy‐induced vascular toxicity: VEGF inhibition and beyond. Int J Cardiol. 2017;227:11‐17. 10.1016/j.ijcard.2016.11.174 27866063

[10] Dong Q , Chen L , Lu Q . Quercetin attenuates doxorubicin cardiotoxicity by modulating Bmi‐1 expression. Br J Pharmacol. 2014;171:4440‐4454. 10.1111/bph.12795 24902966 PMC4209150

[11] Falk R , Falk A , Dyson MR . Generation of anti‐Notch antibodies and their application in blocking Notch signalling in neural stem cells. Methods. 2012;58:69‐78. 10.1016/j.ymeth.2012.07.008 22842086 PMC3502869

[12] Garate‐Carrillo A , Gonzalez J , Ceballos G , Ramirez‐Sanchez I , Villarreal F . Sex related differences in the pathogenesis of organ fibrosis. Transl Res. 2020;222:41‐55. 10.1016/j.trsl.2020.03.008 32289256 PMC7721117

[13] Gieni RS , Ismail IH , Campbell S , Hendzel MJ . Polycomb group proteins in the DNA damage response: a link between radiation resistance and "stemness". Cell Cycle. 2011;10:883‐894. 10.4161/cc.10.6.14907 21346409

[14] Guentchev M , McKay RD . Notch controls proliferation and differentiation of stem cells in a dose‐dependent manner. Eur J Neurosci. 2006;23:2289‐2296. 10.1111/j.1460-9568.2006.04766.x 16706837

[15] Harvey PA , Leinwand LA . Oestrogen enhances cardiotoxicity induced by Sunitinib by regulation of drug transport and metabolism. Cardiovasc Res. 2015;107:66‐77. 10.1093/cvr/cvv152 26009590 PMC4560048

[16] Ismail IH , Andrin C , McDonald D , Hendzel MJ . BMI1‐mediated histone ubiquitylation promotes DNA double‐strand break repair. J Cell Biol. 2010;191:45‐60. 10.1083/jcb.201003034 20921134 PMC2953429

[17] Jabbour E , Kantarjian H . Chronic myeloid leukemia: 2018 update on diagnosis, therapy and monitoring. Am J Hematol. 2018;93:442‐459. 10.1002/ajh.25011 29411417

[18] Kato R , Yamazoe Y . Sex‐specific cytochrome P450 as a cause of sex‐and species‐related differences in drug toxicity. Toxicol Lett. 1992;4274(92):661–667. 10.1016/0378-4274(92)90245-F 1471220

[19] Kim S , You D , Jeong Y , Yoon SY , Kim SA , Lee JE . Inhibition of platelet‐derived growth factor C and their receptors additionally increases doxorubicin effects in triple‐negative breast cancer cells. Eur J Pharmacol. 2021;895:173868. 10.1016/j.ejphar.2021.173868 33460613

[20] Lin KR , Huang JT , Henderson CJ , Wolf CR . Novel Pathways of Ponatinib Disposition Catalyzed By CYP1A1 Involving Generation of Potentially Toxic Metabolites. J Pharmacol Exp Ther. 2017;363:12‐19. 10.1124/jpet.117.243246 28882992 PMC5596814

[21] Lionetti V , Matteucci M , Ribezzo M . Regional mapping of myocardial hibernation phenotype in idiopathic end‐stage dilated cardiomyopathy. J Cell Mol Med. 2014;18:396‐414. 10.1111/jcmm.12198 24444256 PMC3955147

[22] Liu N , Zhang J , Ji C . The emerging roles of Notch signaling in leukemia and stem cells. Biomark Res. 2013;1:23. 10.1186/2050-7771-1-23 24252593 PMC4177577

[23] Lozano GM , Bejarano I , Espino J . Relationship between caspase activity and apoptotic markers in human sperm in response to hydrogen peroxide and progesterone. J Reprod Dev. 2009;55:615‐621. 10.1262/jrd.20250 19734695

[24] Madonna R , Pieragostino D , Cufaro MC . Ponatinib Induces Vascular Toxicity through the Notch‐1 Signaling Pathway. J Clin Med. 2020;9:820. 10.3390/jcm9030820 PMC7141219 32197359

[25] Madonna R , Giovannelli G , Confalone P , Renna FV , Geng YJ , De Caterina R . High glucose‐induced hyperosmolarity contributes to COX‐2 expression and angiogenesis: implications for diabetic retinopathy. Cardiovasc Diabetol. 2016;15:18. 10.1186/s12933-016-0342-4 26822858 PMC4731895

[26] Madonna R , Doria V , Gorbe A . Co‐expression of glycosylated aquaporin‐1 and transcription factor NFAT5 contributes to aortic stiffness in diabetic and atherosclerosis‐prone mice. J Cell Mol Med. 2020;24:2857‐2865. 10.1111/jcmm.14843 31970899 PMC7077545

[27] Neff LS , Bradshaw AD . Cross your heart? Collagen cross‐links in cardiac health and disease. Cell Signal. 2021;79:109889. 10.1016/j.cellsig.2020.109889 33347984 PMC8830414

[28] Park IK , Morrison SJ , Clarke MF . Bmi1, stem cells, and senescence regulation. J Clin Invest. 2004;113:175‐179. 10.1172/JCI20800 14722607 PMC311443

[29] Pelzer T , Schumann M , Neumann M . 17beta‐estradiol prevents programmed cell death in cardiac myocytes. Biochem Biophys Res Commun. 2000;268:192‐200. 10.1006/bbrc.2000.2073 10652235

[30] Pentassuglia L , Graf M , Lane H . Inhibition of ErbB2 by receptor tyrosine kinase inhibitors causes myofibrillar structural damage without cell death in adult rat cardiomyocytes. Exp Cell Res. 2009;315:1302‐1312. 10.1016/j.yexcr.2009.02.001 19331811 PMC4991362

[31] Perez‐Riverol Y , Csordas A , Bai J . The PRIDE database and related tools and resources in 2019: improving support for quantification data. Nucleic Acids Res. 2019;47:D442‐D450. 10.1093/nar/gky1106 30395289 PMC6323896

[32] Pui JC , Allman D , Xu L . Notch1 expression in early lymphopoiesis influences B versus T lineage determination. Immunity. 1999;11:299‐308. 10.1016/s1074-7613(00)80105-3 10514008

[33] Pursglove SE , Mackay JP . CSL: a notch above the rest. Int J Biochem Cell Biol. 2005;37:2472‐2477. 10.1016/j.biocel.2005.06.013 16095948

[34] Radtke F , Fasnacht N , Macdonald HR . Notch signaling in the immune system. Immunity. 2010;32:14‐27. 10.1016/j.immuni.2010.01.004 20152168

[35] Radtke F , Raj K . The role of Notch in tumorigenesis: oncogene or tumour suppressor? Nat Rev Cancer. 2003;3:756‐767. 10.1038/nrc1186 14570040

[36] Ren M , Cowell JK . Constitutive Notch pathway activation in murine ZMYM2‐FGFR1‐induced T‐cell lymphomas associated with atypical myeloproliferative disease. Blood. 2011;117:6837‐6847. 10.1182/blood-2010-07-295725 21527531 PMC3128478

[37] Saadoun S , Papadopoulos MC , Davies DC , Bell BA , Krishna S . Increased aquaporin 1 water channel expression in human brain tumours. Br J Cancer. 2002;87:621‐623. 10.1038/sj.bjc.6600512 12237771 PMC2364235

[38] Saussele S , Haverkamp W , Lang F . Ponatinib in the Treatment of Chronic Myeloid Leukemia and Philadelphia Chromosome‐Positive Acute Leukemia: Recommendations of a German Expert Consensus Panel with Focus on Cardiovascular Management. Acta Haematol. 2020;143:217‐231. 10.1159/000501927 31590170 PMC7384349

[39] Singh AP , Umbarkar P , Tousif S , Lal H . Cardiotoxicity of the BCR‐ABL1 tyrosine kinase inhibitors: Emphasis on ponatinib. Int J Cardiol. 2020;316:214‐221. 10.1016/j.ijcard.2020.05.077 32470534 PMC8095092

[40] Tan FH , Putoczki TL , Stylli SS , Luwor RB . Ponatinib: a novel multi‐tyrosine kinase inhibitor against human malignancies. Onco Targets Ther. 2019;12:635‐645. 10.2147/OTT.S189391 30705592 PMC6343508

[41] Trivedi PP , Kushwaha S , Tripathi DN , Jena GB . Cardioprotective effects of hesperetin against doxorubicin‐induced oxidative stress and DNA damage in rat. Cardiovasc Toxicol. 2011;11:215‐225. 10.1007/s12012-011-9114-2 21553131

[42] Wu S , Ko YS , Teng MS . Adriamycin‐induced cardiomyocyte and endothelial cell apoptosis: in vitro and in vivo studies. J Mol Cell Cardiol. 2002;34:1595‐1607. 10.1006/jmcc.2002.2110 12505058

[43] Yin DD , Fan FY , Hu XB . Notch signaling inhibits the growth of the human chronic myeloid leukemia cell line K562. Leuk Res. 2009;33:109‐114. 10.1016/j.leukres.2008.06.023 18687467

[44] Rocca C , Femmino S , Aquila G , et al. Notch1 Mediates Preconditioning Protection Induced by GPER in Normotensive and Hypertensive Female Rat Hearts. Front Physiol. 2018;9(521):2018. 10.3389/fphys.2018.00521 PMC5962667 29867564

[45] Caliceti C , Aquila G , Pannella M , et al. 17beta‐estradiol enhances signalling mediated by VEGF‐A‐delta‐like ligand 4‐notch1 axis in human endothelial cells. PLoS One. 2013;8:e71440. 23967210 10.1371/journal.pone.0071440 PMC3742772

