# Supplementary figures and images for: Sex‐related differential susceptibility to ponatinib cardiotoxicity and differential modulation of the Notch1 signalling pathway in a murine model
Source: J Cell Mol Med. 2022 Feb 5;26(5):1380–91. doi: 10.1111/jcmm.17008 (PMC8899159; doi:10.1111/jcmm.17008)

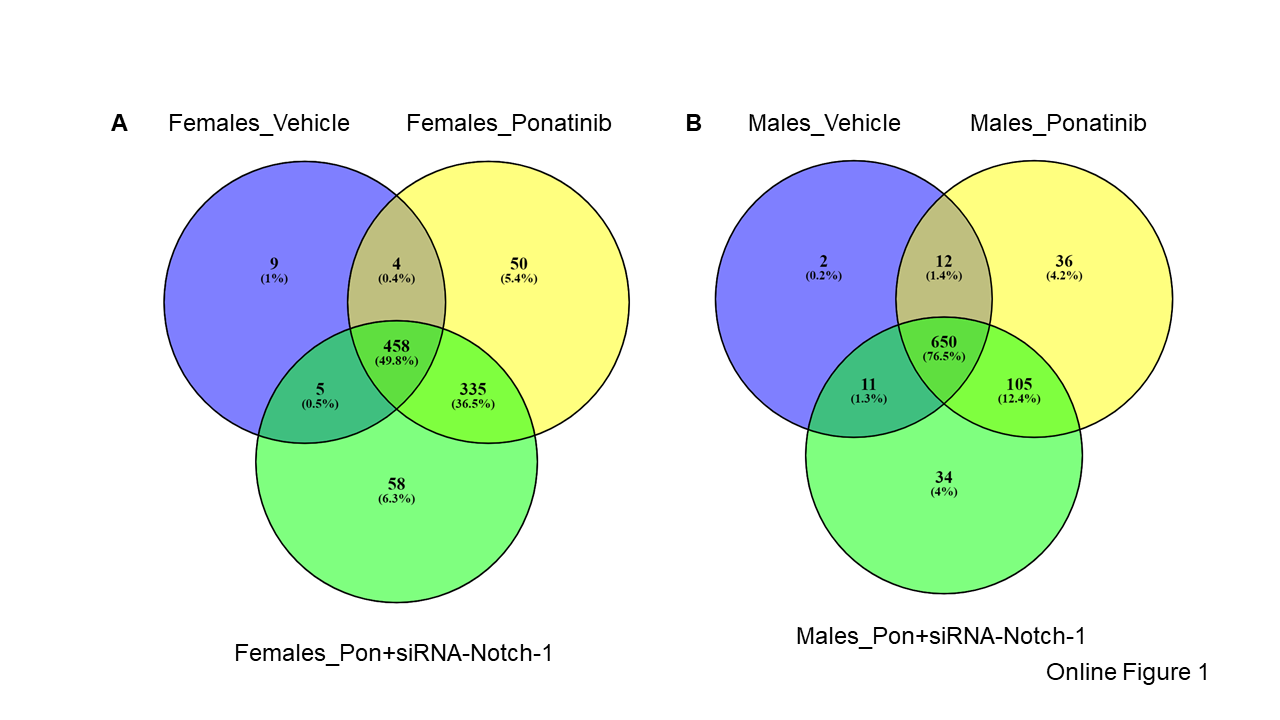

Supplement: Supplementary file 1 — Fig S1 [file JCMM-26-1380-s004.tif]

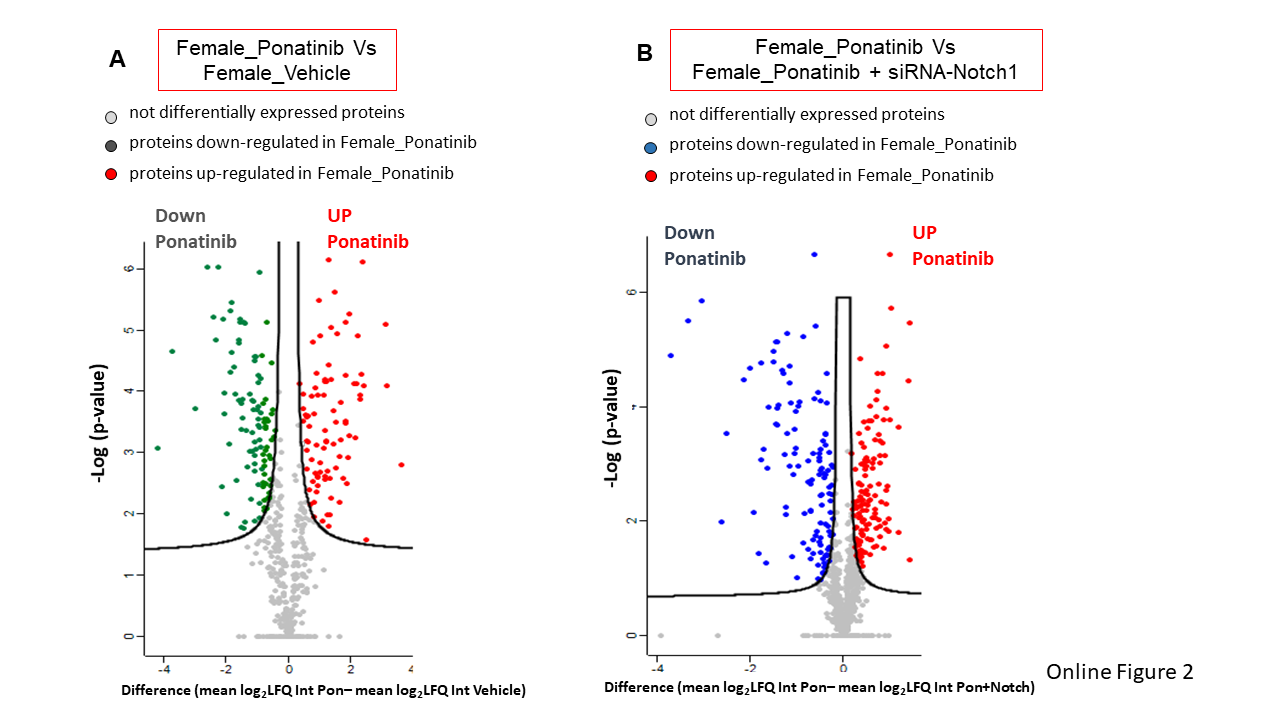

Supplement: Supplementary file 2 — Fig S2 [file JCMM-26-1380-s005.tif]

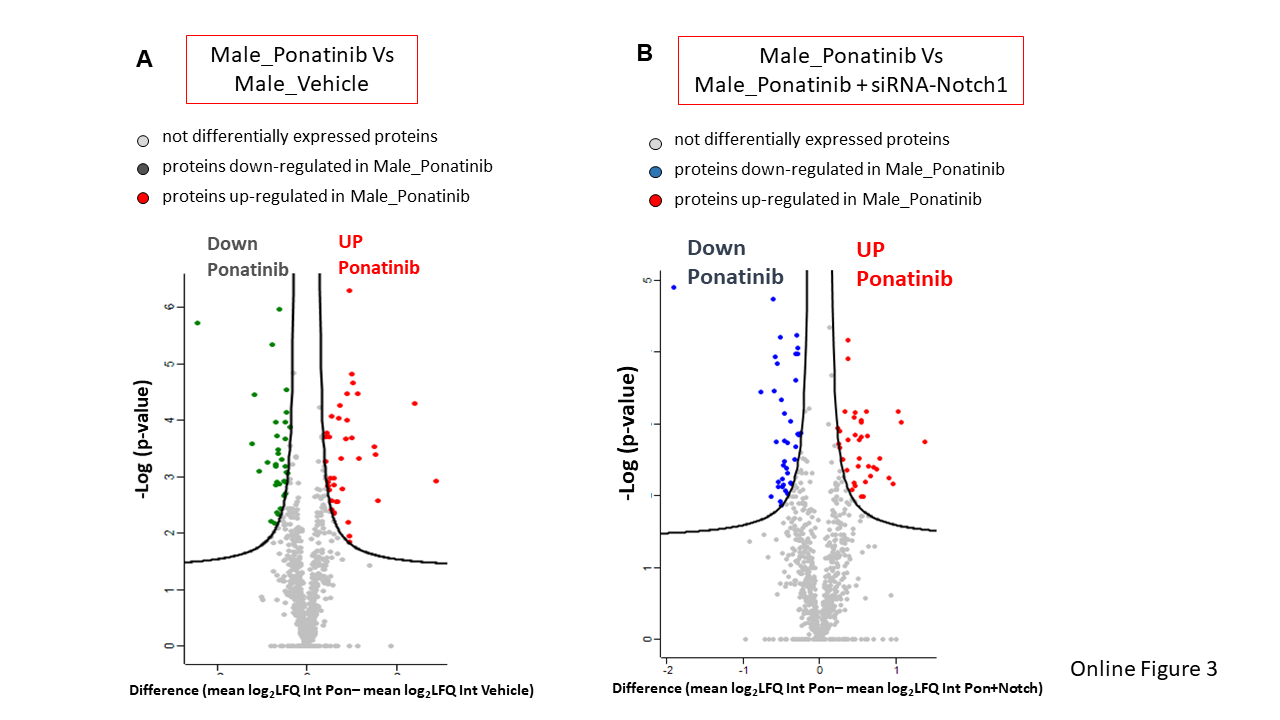

Supplement: Supplementary file 3 — Fig S3 [file JCMM-26-1380-s003.tif]

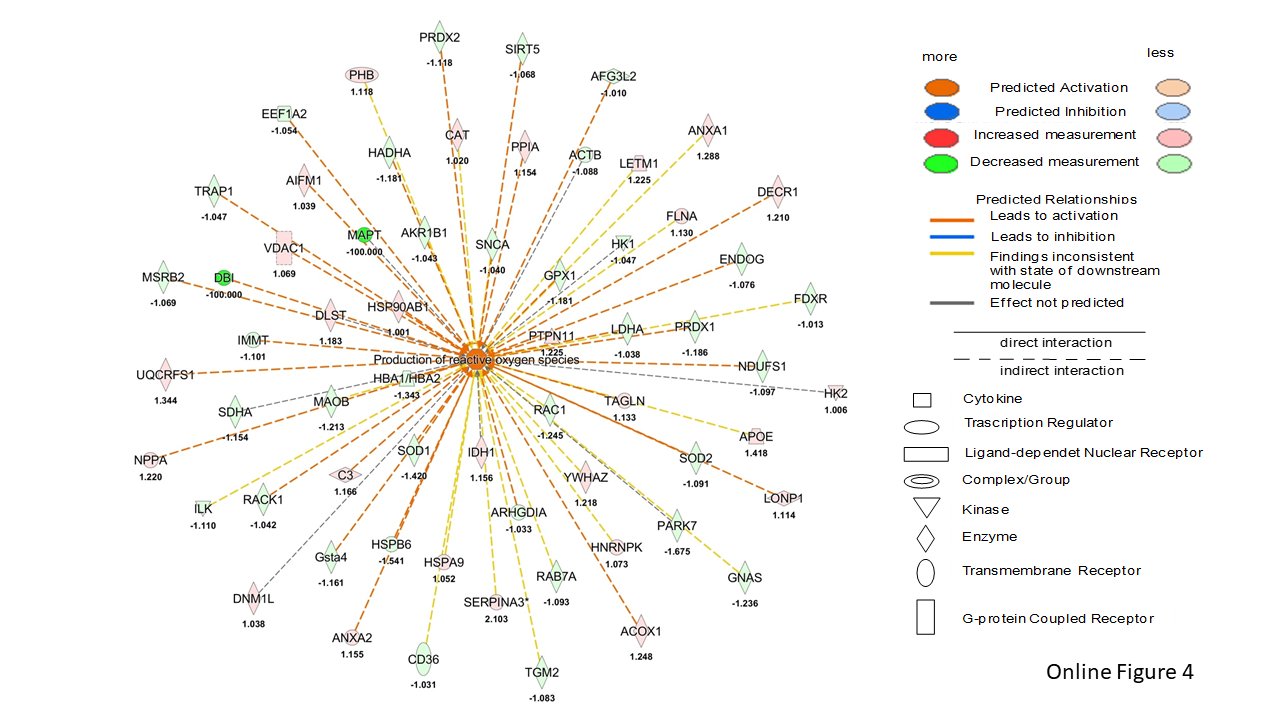

Supplement: Supplementary file 4 — Fig S4 [file JCMM-26-1380-s001.tif]

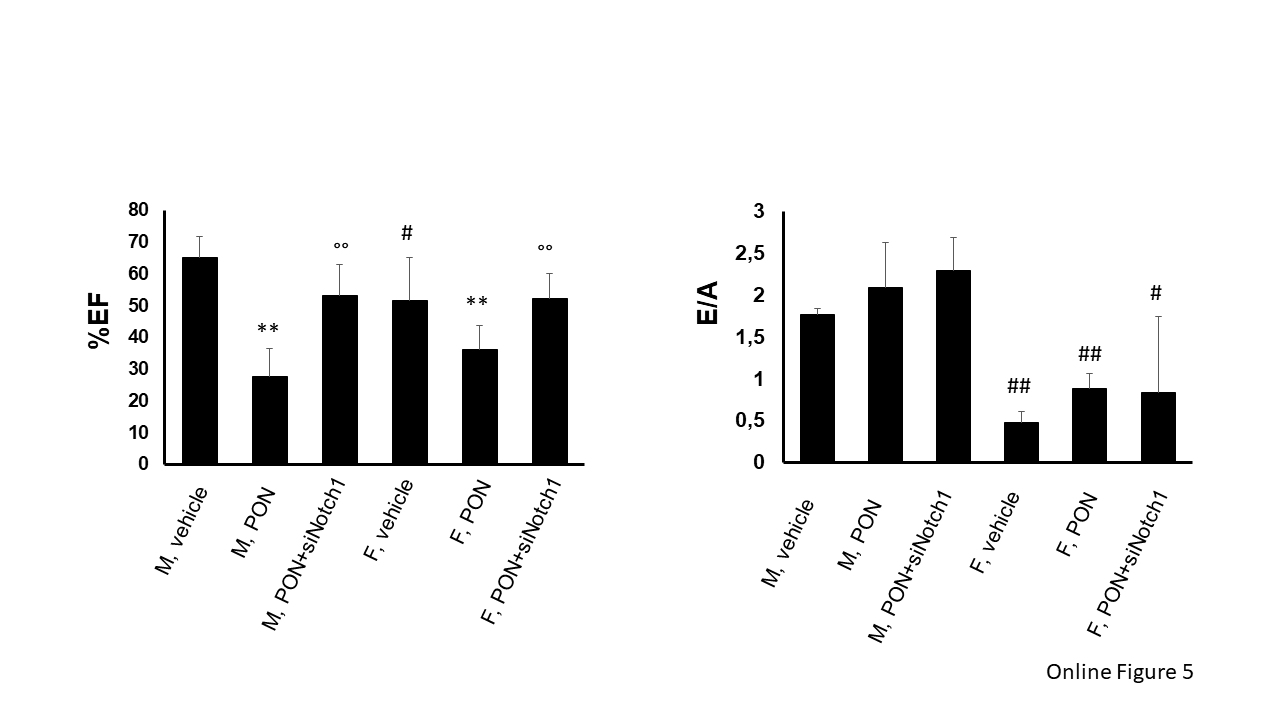

Supplement: Supplementary file 5 — Fig S5 [file JCMM-26-1380-s002.tif]
