# Supplementary material for: Sex‐related differential susceptibility to ponatinib cardiotoxicity and differential modulation of the Notch1 signalling pathway in a murine model
Source: J Cell Mol Med. 2022 Feb 5;26(5):1380–91. doi: 10.1111/jcmm.17008 (PMC8899159; doi:10.1111/jcmm.17008)
Supplement: Supplementary file 6 — Supplementary Material [file JCMM-26-1380-s006.docx]

**Sex-related differential susceptibility to ponatinib cardiotoxicity and differential modulation of the Notch1 signaling pathway in a murine model**

Rosalinda Madonna^1*^, Damiana Pieragostino^2,4*^, Maria Concetta Cufaro^3,4^, Piero Del Boccio^3,4^, Angela Pucci^5^, Letizia Mattii^6^, Vanessa Doria^7^, Christian Cadeddu Dessalvi^8^, Riccardo Zucchi^9^, Giuseppe Mercuro^8^, Raffaele De Caterina^1,10^

*co-first author

^1^ Institute of Cardiology, University of Pisa, Pisa, Italy

^2^ Department of Innovative Technologies in Medicine and Dentistry, ‘‘G. d’Annunzio’’ University of Chieti-Pescara, Chieti, Italy

^3^ Department of Pharmacy, ‘‘G. d’Annunzio’’ University of Chieti-Pescara, Chieti, Italy

^4^ Analytical Biochemistry and Proteomics Laboratory, Center for Advanced Studies and Technology (CAST), "G. d'Annunzio" University of Chieti-Pescara, Italy

^5^ Department of Histopathology, Pisa University Hospital, Italy

^6^ Department of Clinical and Experimental Medicine, University of Pisa, Pisa, Italy

^7^ Institute of Cardiology, “G. D’Annunzio” University of Chieti-Pescara Italy

^8^ Department of Medical Sciences and Public Health, University of Cagliari, Italy

^9^ Department of Pathology, Laboratory of Biochemistry, University of Pisa, Italy

^10^ Fondazione VillaSerena per la Ricerca, Città Sant’Angelo, Pescara, Italy

**Online Supplement**

**SUPPLEMENTAL MATERIAL AND METHODS**

**Label Free Proteomics Analysis** *-* To analyze the effects of PON with/without siRNA-Notch1 or siRNA-scrambled on the specific expressional signatures in cardiac tissue, shotgun proteomics analyses were performed. Cardiac tissues from each treatment group were prepared according to the Filter Aided Sample Preparation (FASP) method. Briefly, cardiac tissues were lysed by sonication in RIPA buffer and centrifuged to remove cell debris. Protein concentration was measured by Bradford assay (Bio-Rad, Hercules, CA, USA) using Bovine Serum Albumin (BSA, Sigma-Aldrich, St. Louis, MI, USA) standards for the calibration curve. 50 µg of proteins for each treatment were digested by trypsin (Promega, Madison, WI, USA). For protein label free identification and quantification, tryptic peptides from each sample were analyzed in triplicate with LC-MS/MS using the UltiMate^TM^ 3000 UPLC (Thermo Fisher Scientiﬁc) chromatographic system coupled to the Orbitrap Fusion^TM^ Tribrid^TM^ (Thermo Fisher Scientiﬁc) mass spectrometer. Peptides were loaded on the Trap Cartridge C18 (0.3 mm ID, 5 mm L, 5 μm PS, Thermo Fisher Scientific) and then separated on an EASY-spray Acclaim^TM^ PepMap^TM^ C18 (75 μm ID, 25 cm L, 2 μm PS, Thermo Fisher Scientific) nanoscale chromatographic column. Mobile phase A was 0.1% formic acid in H_2_O and mobile phase B was 0.1% formic acid in acetonitrile. The flow rate was set at 300 nL/min, with a total run time of 120 minutes using a chromatographic gradient from 2% to 90% of phase B. Proteomics data were acquired in positive-ion polarity with Data Dependent Acquisition (DDA) mode to trigger precursor isolation and MS2 sequence using N_2_ as collision gas for CID fragmentation. Positive ion voltage was set at 1700 V and the ion transfer tube at 250 °C for desolvation. MS1 scans were performed in the Orbitrap analyzer covering a m/z range of 375-1500 with 120,000 of resolution. A standard automatic gain control (AGC) target and an automatic maximum injection time (MIT) were used. The signal intensity threshold was set to 5x10^3^ and the MS2 spectra were acquired using a Top Speed method of 3s. In particular, precursor ions with charges of +2 to +7 were used for MS2 sequencing and scanned in the ion trap by setting the following parameters: MS2 isolation window of 1.6 Da, AGC target of 2x10^3^, dynamic exclusion time of 60s and mass tolerance of ±10 ppm were used. We performed a CID fragmentation with a fixed collision energy of 35% and an activation time of 10ms.

**Proteomics Data Processing** *-* Proteomics raw data were processed using a free computational platform, MaxQuant version 1.6.6.0 (Max-Planck Institute for Biochemistry, Martinsried, Germany). Peak lists, generated in MaxQuant, were searched using Andromeda peptide search engine against the UniProt database (released 2019_11, taxonomy *Mus Musculus*, 21,990 entries) supplemented with frequently observed contaminants and containing forward and reverse sequences. As previously reported [1,2], carbamidomethylation of cysteines (C) was defined as fixed modification, while oxidation of methionines (M), deamidation of asparagines (N) and glutamines (Q) were set as variable modifications. False discovery rate (FDR) both at the protein level and at peptide level were set at 1%. LFQ Intensity [3] was used to quantify protein abundance in each sample. Bioinformatics analysis was performed with Perseus version 1.6.10.50 (Max-Planck Institute for Biochemistry, Martinsried, Germany). LFQ intensities were log_2_ transformed to facilitate the calculation of the protein expression. Site only, reverse and contaminant peptides were removed from the dataset. The minimum number of valid values accepted was set at 2 in at least one group. In this way we evaluated not only the different protein expression, but also the presence and absence of proteins between the different treatment conditions. Moreover, an univariate statistical analysis was performed with a p-value threshold of 0.05 in order to define the significantly differential proteins between cardiac tissues of female and male mice treated with PON+siRNA-scrambled with those treated with vehicle or with PON+siRNA-Notch1. Results were visualized as Volcano Plots (Online Figure 2 and Online Figure 3).

At last, differentially expressed proteins were uploaded for “Core Analysis” through Ingenuity Pathway Analysis tool, (IPA, Qiagen, Hilden, Germany). IPA is able to map statistically the modulated proteins for their functional annotations, such as Canonical Pathways, Upstream Regulators Analysis and downstream effects networks, through Gene Ontology and pathway analysis. In this way, it is possible to identify the metabolic pathways and the secondary genes/proteins inhibited (z-score ≤ -2.00) and/or stimulated (z-score ≥ 2.00) for a specific phenotype and consequently classify potential effectors molecules and/or a pharmacological target.

**Echocardiography** - Using a portable ultrasound apparatus (Esaote; Genoa, Italy) equipped with a 21-MHz linear probe, we performed transthoracic echocardiography 28 days after treatments to assess the functional effects of each treatment. The mice were anesthetized (ketamine, 100 mg/kg) and placed in the left lateral decubitus position. Two-dimensional (B-mode) and mono-dimensional mode (M-mode) images of parasternal short-axis and long-axis views were acquired to position the Motion-mode (M-mode) cursor at the level of the papillary muscles and perpendicular to the interventricular septum and LV free-wall. To evaluate LV structural changes, several parameters from M-mode were measured (i.e., LV end-diastolic diameter [LVEDD] and LV end-systolic diameter [LVESD]). Left ventricular fractional shortening (FS%) was calculated as: FS (%) = ([LVEDD – LVESD)/LVEDD] × 100. Left ventricular ejection fraction (EF%) was calculated as an index of systolic function: EF (%) = ([LVEDD_3_ – LVESD_3_)/LVEDD_3_] × 100. The same parameters were measured on control, non-manipulated healthy animals. Passive LV filling peak velocity (*E*, mm/s) and atrial contraction flow peak velocity (*A*, mm/s) were obtained by Pulsed-wave Doppler through the mitral valve. Each measurement was obtained by averaging the results of 3 consecutive heart beats. Individuals conducting the echocardiography were blinded to the animal treatment groups.

**SUPPLEMENTAL REFERENCES**

1. Brocco D, Lanuti P, Pieragostino D (2021). Phenotypic and Proteomic Analysis Identifies Hallmarks of Blood Circulating Extracellular Vesicles in NSCLC Responders to Immune Checkpoint Inhibitors. Cancers (Basel);13.

2. Falasca K, Lanuti P, Ucciferri C (2021). Circulating extracellular vesicles as new inflammation marker in HIV infection. AIDS 35:595-604.

3. Cox J, Hein MY, Luber CA, Paron I, Nagaraj N, Mann M (2014). Accurate proteome-wide label-free quantification by delayed normalization and maximal peptide ratio extraction, termed MaxLFQ. Mol Cell Proteomics;13:2513-2526.

**LEGEND TO SUPPLEMENTAL FIGURES**

**Online Figure 1:** Venn diagram of identified and quantified proteins in each samples: female cardiac tissue (panel A) and male cardiac tissue (panel B).

**Online Figure 2: Differential proteomics analysis of female mice heart tissue.**

Volcano Plot in panel A shows differentially proteins expressed in mice treated with PON+siRNA- scrambled compared to those treated with only vehicle, whereas panel B shows differentially proteins expressed in mice treated with PON+siRNA- scrambled compared to those co-treated with PON+siRNA-Notch1. Protein expression legend is reported in the figure. In both cases proteins were graphed by fold change (Difference) and -Log(P value).

**Online Figure 3: Differential proteomics analysis of male mice heart tissue.**

Volcano Plot in panel A shows differentially proteins expressed in mice treated with PON+siRNA- scrambled compared to those treated with only vehicle, whereas panel B shows differentially proteins expressed in mice treated with PON+siRNA- scrambled compared to those co-treated with PON+siRNA-Notch1. Protein expression legend is reported in the figure. In both cases proteins were graphed by fold change (Difference) and -Log(P value).

**Online Figure 4:** Downstream network effects of quantified protein in Ingenuity Pathway Analysis (IPA) revealed the activation of “production of reactive oxygen species” in male mice cardiac tissue treated with PON+siRNA- scrambled compared to those co-treated with PON+siRNA-Notch1**.** Red and green shapes represent increased or decreased measurements of identified proteins, respectively. Color intensity is directly proportional to the fold change value of each protein reported in the figure. Color key and symbols are reported in the legend.

**Online Figure 5: Effects of ponatinib and Notch-1 signaling inhibition on cardiac function in male and female mice.** Panels A, Ejection Fraction (%EF) and B, E/A ratio measured by echo-doppler in the different treatment groups such as control (vehicle), PON+siRNA-scrambled -treated mice, and PON+siRNA-Notch1 treated mice. Data are expressed as means ± standard deviations (n=6 mice per treatment group). ** vs vehicle, p < 0.01; °° vs PON+siRNA-Notch1, p<0.01; # vs M, p<0.05; ## vs M, p<0.01. Abbreviations: PON, ponatinib; M, male; F, female.
